# Differential Expression of Tissular miRNA-155 in Pediatric Gastritis

**DOI:** 10.3390/jcm11123351

**Published:** 2022-06-10

**Authors:** Săsăran Maria Oana, Bănescu Claudia, Riza Anca Lelia, Mocan Simona, Cârstea Claudia, Dobru Ecaterina Daniela

**Affiliations:** 1 Department of Pediatrics III, George Emil Palade University of Medicine, Pharmacy, Science and Technology of Targu Mures, Gheorghe Marinescu Street No 38, 540136 Targu Mures, Romania; oanam93@yahoo.com; 2 Genetics Department, Center for Advanced Medical and Pharmaceutical Research, George Emil Palade University of Medicine, Pharmacy, Science and Technology of Targu Mures, Romania, Gheorghe Marinescu Street No 38, 540136 Targu Mures, Romania; clau_genetic@yahoo.com; 3 Laboratory of Human Genomics, University of Medicine and Pharmacy of Craiova, Petru Rareș Street No 2, 200349 Craiova, Romania; anca_riza@yahoo.com; 4 Pathology Department, County Emergency Clinical Hospital of Targu Mures, Gheorghe Marinescu Street No 50, 540136 Targu Mures, Romania; slmocan@yahoo.com; 5 Department of Internal Medicine VII, George Emil Palade University of Medicine, Pharmacy, Science and Technology of Targu Mures, Gheorghe Marinescu Street No 38, 540136 Targu Mures, Romania; danidobru@gmail.com

**Keywords:** pediatric gastritis, miR-155, biomarker, *Helicobacter pylori*, chronic gastritis

## Abstract

Background: MicroRNA molecules, among them the intensely studied miRNA-155 (miR-155), are regarded as potential biomarkers of chronic gastric inflammation and premalignant lesion progression. However, literature data are scarce in terms of pediatric studies and in the evaluation of the predictive role of miRNA in early gastric inflammation. This study aims to assess the differential expression of miR-155 in relation to pediatric gastritis. Methods: The present research was conducted on 192 patients with chronic dyspeptic symptoms who underwent upper digestive endoscopy. Bioptic samples were harvested for histopathological analysis and tissue miR-155 depiction. MiR-155 expression analysis was carried out through quantitative real-time polymerase chain reaction (qRT-PCR). The study population was divided into two groups: controls (93 patients) and study group (99 patients) with inflammatory modifications. Results: MiR-155 expression was augmented in patients with gastritis but did not differ significantly from controls (*p* = 0.16). An increase in miR-155 expression was noted in relation to chronic gastritis, *H. pylori* infection, or increase in gastritis severity, but these variations were not important (*p* = 0.30, *p* = 0.44, and *p* = 0.45, respectively). Conclusions: According to our study, pediatric gastritis increases, but does not greatly influence, miR-155 expression. Dynamic evaluation of miR-155 might enlighten its prognostic role in pediatric gastritis.

## 1. Introduction

Gastritis represents one of the major public health concerns in children, with particularities in terms of histology findings and comprising a large spectrum of dyspeptic symptoms [1]. *Helicobacter pylori* (*H. pylori*) remains the most frequently encountered etiological factor of pediatric gastritis and can persist until adulthood, being responsible for chronic inflammation of the gastric mucosa, as well as for digestive and extra-digestive complications [2]. Considered a major carcinogenetic factor, *H. pylori*-induced chronic inflammatory modification of the gastric mucosa can evolve into preneoplastic lesions, which become more prevalent with age increase [3]. Ultimately, the immune response will be essential for infection progression and initiation of carcinogenetic processes, which imply the activation of numerous molecular pathways, resulting in alterations of cellular deoxyribonucleic acid (DNA) and ribonucleic acid (RNA) [4,5].

Recent studies have focused upon molecular modulators of signaling pathways involved in gastric inflammation processes. These have included investigations upon the role of miRNAs, short non-coding RNAs which negatively regulate gene expression at the post-transcriptional level [6,7,8]. One of the main functions of miRNAs is the downregulation of protein synthesis, including the ones responsible for cell division inhibition. Thus, alteration of miRNA expression has been linked to abnormal cell division, an important milestone in the initiation and evolution of carcinogenesis [9,10]. MiRNAs have, however, been proven to play a role in various biologic processes and their dependence upon target gene expression translates into contradictory effects on different organs and tissues [11]. Given their proven role in the modulation of the innate and adaptative immune response, miRNAs are regarded as key regulators of inflammatory pathways triggered by pathogens such as *H. pylori* [6,12].

MiRNA-155 (miR-155) has been one of the most intensively studied miRNAs, with active implications in both physiological and pathological processes, and emerged as a marker of chronic gastric inflammation, possibly predictive of oncogenesis [13,14]. Its potential biomarker trait in gastritis and gastric cancer originated from studies supporting its immunomodulatory role. MiR-155 expression is mainly augmented by activation of B and T lymphocytes, which explains its reported upregulation in relation to various conditions [15]. Furthermore, in vitro studies proved the lack of an adequate immune response in subjects with absent or minimal miR-155 expression [16,17]. MiR-155 also stimulates differentiation of T helper 17 (Th17) lymphocytes, key effectors of *H. pylori*-associated immune response and inflammation. The presence of the pathogen within the gastric mucosa has been associated with the augmentation of miR-155 expression, a consequence of toll-like receptor (TLR) activation and the release of tumor necrosis factor-alpha (TNF-α) [18]. Adequate expression of miR-155 is also mandatory to prevent excessive inflammatory responses triggered by *H. pylori* infection, with miR-155 acting as an integrated part of a negative feedback loop mechanism which will, in the end, inhibit the release of pro-inflammatory cytokines [12,19].

Considering the capacity of miRNAs to modulate host immune response resulting from pathogenic exogenous stimuli, miRNA expression alteration in chronic gastritis and gastric cancer has been assessed mostly in association with *H. pylori* infection [20]. Moreover, there are very few available data on the role of miRNA as a biomarker of acute gastritis, with most of the research conducted so far focusing mainly on progressive expression alterations related to gastric carcinogenesis cascades [14,21,22]. Furthermore, there is currently only one study available in the literature which analyzed the expression variation of two miRNAs in pediatric gastritis, namely miR-155 and miR-146a [23].

*The main objective* of the current study is to assess the differential expression of miR-155 in relation to pediatric gastritis. Considering the wide number of pediatric patients with gastritis of non-*H. pylori* etiologies, as well as the limited knowledge on the subject, this study aimed to assess whether miR-155 expression was dependent upon the presence of *H. pylori* in children suffering from gastritis, the types of inflammatory modifications of the gastric mucosa, or upon gastritis severity, established in accordance with the updated Sydney criteria [24].

## 2. Materials and Methods

### 2.1. Study Population

The study enrolled 198 patients aged between 1 and 17 years, admitted to a tertiary pediatric clinic from Târgu Mureş, România between February 2018 and February 2021. *Inclusion criteria* consisted of chronic dyspeptic symptoms (such as abdominal pain, nausea, vomiting, pyrosis), with an indication of upper digestive endoscopy (esophagogastroduodenoscopy (EGD)). In those cases presenting with symptoms which met the Rome IV criteria, suggestive of a diagnosis of functional gastrointestinal disorders, the conductance of an EGD was established upon the positive anamnesis of alarming symptoms (persistent vomiting, dysphagia, odynophagia, hematemesis, melena, involuntary weight loss) or personal/family history of celiac disease, inflammatory bowel disease (IBD), or peptic ulcer [25]. *Exclusion criteria* consisted of viral, bacterial (except for *H. pylori),* and parasitic infections which could have been responsible for the dyspeptic symptoms. In order to rule out these infectious causes, a thorough anamnesis was conducted in order to appropriately assess the onset and evolution of the symptoms, and a parasitological stool examination was performed on those subjects with a suggestive clinical presentation. Furthermore, patients with previously known chronic conditions, such as autoimmune or hematological disorders, malignancies, chronic kidney disease, diabetes, IBD, or celiac disease were also excluded from the study. Patients whose caregivers refused to sign the informed consent prior to participation in the study were also left out of the study population.

### 2.2. Histopathological Analysis and Division of the Study Groups

An upper digestive endoscopy was conducted in each patient by a singular, trained endoscopist, using an Olympus GIF P30 endoscope after a fasting period of at least 8 h. Bioptic samples of the gastric mucosa were harvested from the corporeal and antral gastric mucosa for histopathological analysis (in accordance with the latest guideline criteria for diagnosis of *H. pylori* infection in children and adolescents [26]), as well as for tissue miR-155 expression assessment (two bioptic samples taken from the antral gastric mucosa and one bioptic sample from the corporeal gastric mucosa, from apparent macroscopic changes of the gastric mucosa).

Division of the study groups was based upon the histopathological analysis. Giemsa staining was used for the identification of a concurrent *H. pylori* infection. Assessment of gastritis severity was based upon the updated Sydney classification [24], which focuses on the following parameters: type of inflammatory modifications of the gastric mucosa, lesional activity (assessed in accordance with neutrophil infiltration degree), as well as dysplastic changes of the gastric mucosa (gastric atrophy/intestinal metaplasia). Thus, the control group consisted of children without any pathological modifications of the gastric mucosa, whereas the study group, comprising of patients with inflammatory changes of the gastric mucosa, was further divided according to the following criteria: depiction of *H. pylori* infection, inflammation type (acute/chronic), and severity degree, in accordance with the updated Sydney criteria (mild, moderate and severe).

### 2.3. Assessment of miR-155 Expression

The miRNA expression analysis benefited from the technical support of the infrastructure of the Center for Advanced Medical and Pharmaceutical Research of the George Emil Palade University of Medicine, Pharmacy, Science, and Technology of Târgu Mureş, România. Bioptic samples were preserved and transported on RNA later solution. MiRNA isolation was conducted in accordance with the instructions provided by the phenol-based *mir*Vana™ miRNA Isolation Kit (Thermo Fischer Scientific, Waltham, MA, USA). Complementary DNA (cDNA) was synthesized with the help of a TaqMan MicroRNA Reverse Transcription Kit (Thermo Fischer Scientific, Waltham, MA, USA) and TaqMan miRNA primers. Quantitative real-time PCR (qRT-PCR) was afterward performed with the help of real-time PCR 7500 Fast Dx equipment (Applied Biosystem, Waltham, MA, USA). For qRT-PCR, we used TaqMan^®^ Universal Master Mix II no UNG and a TaqMan miRNA kit (hsa-miR-155*, ID 002287, Thermo Fisher Scientific, Waltham, MA, USA), which included primers and TaqMan hybridization probe. A small nucleolar RNA, RNU48, was used as a reference for normalization, acting as an endogenous control.

The miR-155 expression level was calculated through a comparison with the endogenous control, RNU48, using the delta (Δ) Ct method, with Ct (cycle threshold) representing the minimal number of cycles required to obtain a fluorescent signal within qRT-PCR reactions. Therefore, ΔCt was obtained by calculating the difference between Ct values of miR-155 and those of RNU48. The 2^−ΔCt^ method was subsequently applied by referring to the mean Ct value of the control group, given the absence of paired data [27].

### 2.4. Statistical Analysis

Statistical analysis was conducted using GraphPad Prism T 9.0 software. Descriptive statistics were expressed through parameters such as mean, median, frequency, and standard deviation. Binary variable assessment required the use of the chi-square test. The Shapiro–Wilk normality test evaluated the distribution of analyzed data. Mean comparison of unpaired data was conducted using the Mann–Whitney test (for variables non-compliant with a Gaussian distribution) and the *t*-Student test (for variables complying with a Gaussian distribution). Analysis of variance (ANOVA) and Kruskal–Wallis tests compared the mean and median of at least three data sets. *p* values of under 0.05 were considered statistically significant, corresponding to a confidence interval of 95%.

### 2.5. Ethics

The entire research was conducted in accordance with the principles of the Declaration of Helsinki. A signed informed consent was provided by the legal guardians of each child included in the study. The research protocol was approved by the Ethical Committee of the George Emil Palade University of Medicine, Pharmacy, Science, and Technology of Târgu Mureş, România (Institutional Review Board Approval No. 64/2018 and 507/2019).

## 3. Results

In all, 4 cases out of the total number of 198 were removed from the study population due to alteration of the bioptic samples. Two cases were also excluded due to a microscopic diagnosis of intestinal metaplasia, as the reduced number of patients with dysplastic changes of the gastric mucosa could not have provided a valid study group. Therefore, the final study population consisted of 192 patients. Controls represented 93 patients, whereas inflammatory changes of the gastric mucosa were histologically identified in 99 children.

### 3.1. Expression of miR-155 in Gastric Inflammation

There were no significant differences in terms of age (*p* = 0.09), nor in terms of sex (*p* = 0.6), between the control group and patients diagnosed with any type of gastric inflammation (Table 1). However, a rural environment proved to be a risk factor for gastritis, with an odds ratio (OR) of 2.89 (*p* < 0.01, Table 1).

**Figure 1:**
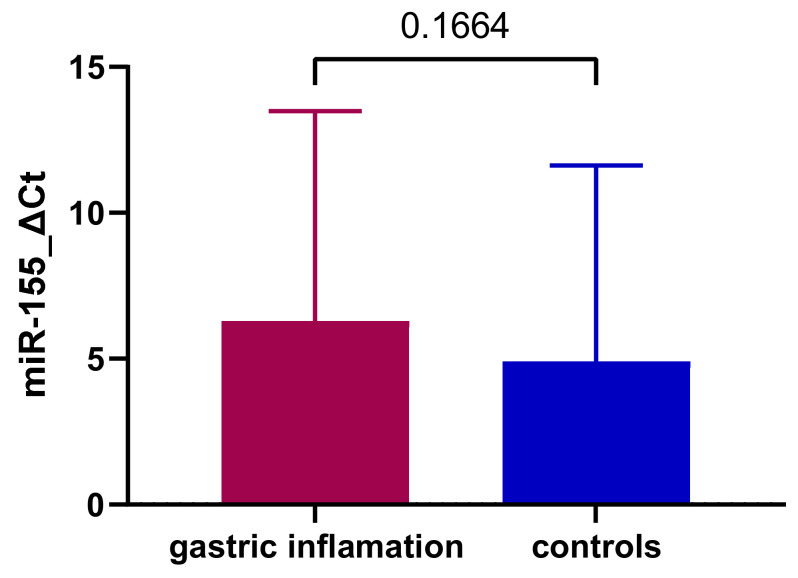
MiR-155 expression comparison between patients with gastritis and control group.

MiR-155 expression presented higher mean values in children with gastric inflammation but without a significant variation from controls (6.29 ± 7.2 SD versus 4.9 ± 6.72 SD, *p* = 0.16, Figure 1).

### 3.2. Expression of miR-155 in Gastric Inflammation Associated with H. pylori Infection

*H. pylori* infection was identified in 39 patients, which represented 20.31% of the entire study population. Furthermore, this number of patients diagnosed with *H. pylori* accounted for 39.39% of patients diagnosed with gastritis. A demographic comparison of the two study groups, divided dependently on the presence of *H. pylori* infection, is enlisted in Table 2. A rural environment proved once again to be a risk factor for the development of gastritis, the association being particularly significant with the concomitant diagnosis of *H. pylori* infection.

**Figure 2:**
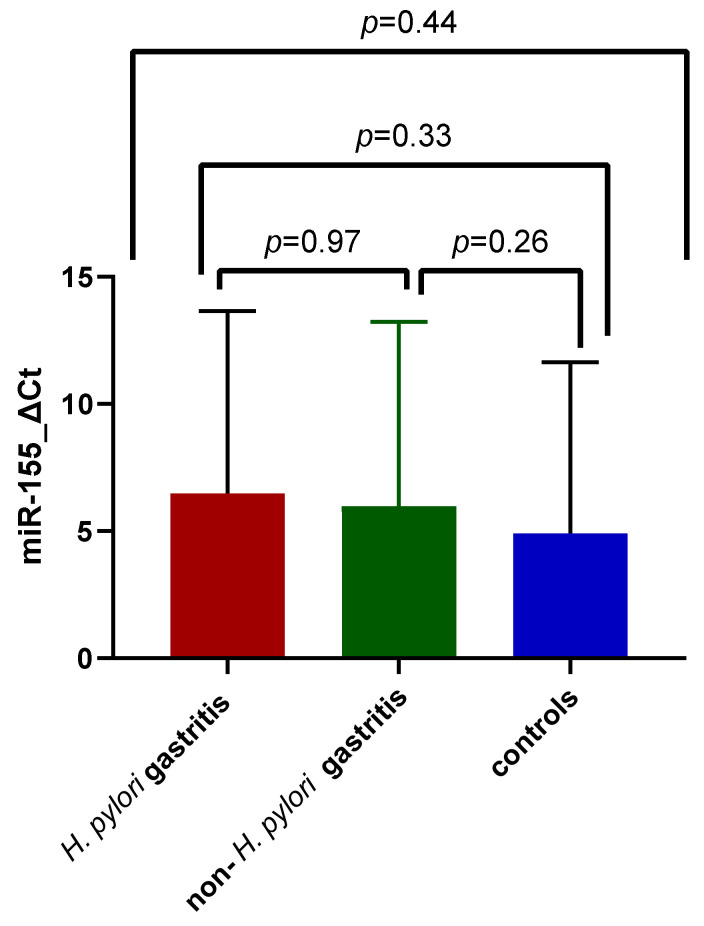
MiR-155 expression comparison between patients with *H. pylori* gastritis, non-*H. pylori* gastritis, and control group.

The highest miR-155 expression was identified among subjects diagnosed with *H. pylori* gastritis (6.47 ± 7.18 SD, versus 5.97 ± 7.24 SD and 4.9 ± 6.72 SD), but without a significant difference from the non-*H. pylori* gastritis or control group mean values, as illustrated in Figure 2. The Kruskal–Wallis test was applied to assess mean differences among the three study groups (*p* = 0.44).

**Figure 3:**
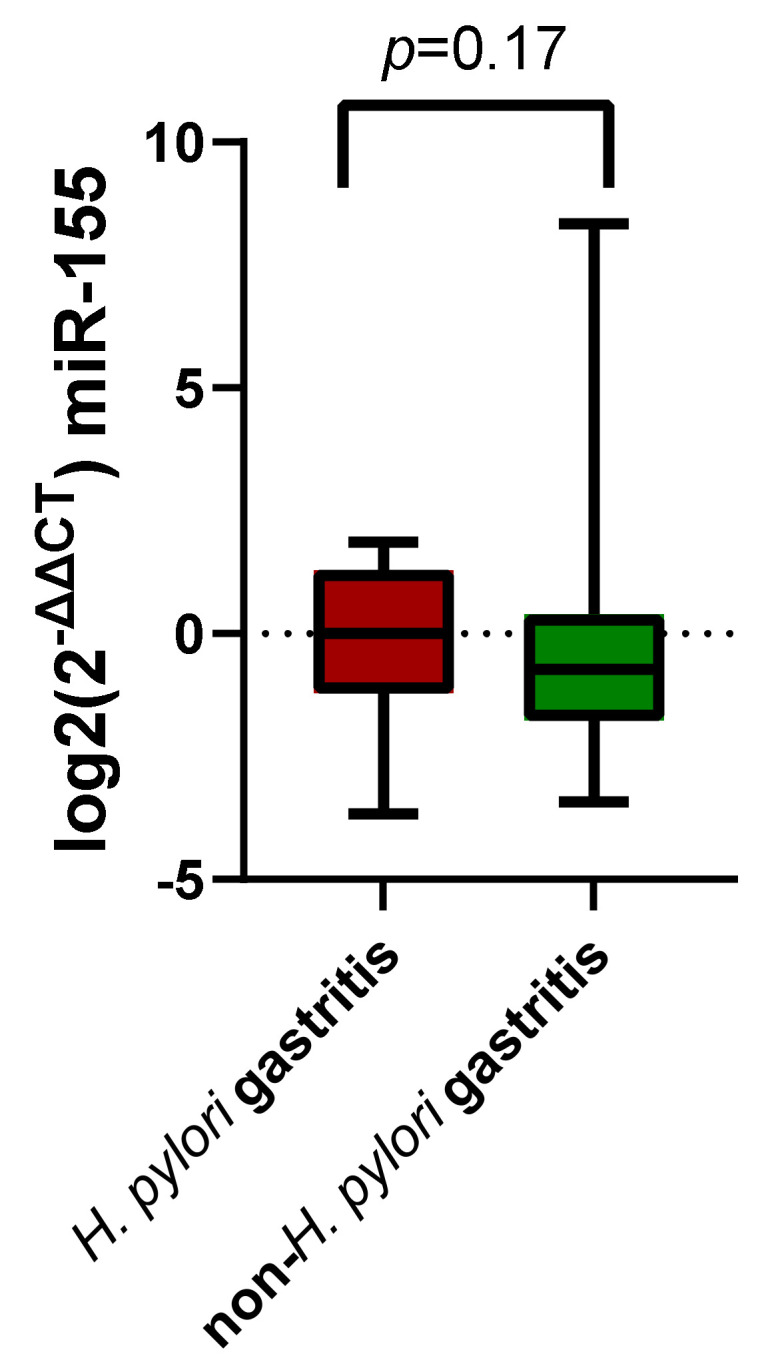
Log_2_(2^−ΔΔCt^) miR-155 comparison between patients with *H. pylori* gastritis and non-*H. pylori* gastritis.

Another comparison was conducted between the two study groups, divided depending on *H. pylori* infectious status, after applying the 2^−ΔΔCt^ method. In spite of a mean upregulation of miR-155 expression within the *H. pylori* gastritis subjects, this increase in miR-155 expression was not statistically significant (Figure 3, *p* = 0.17).

### 3.3. Expression of miR-155 in Acute and Chronic Gastric Inflammation

The study group was divided into two subgroups in accordance with the histopathological description: namely, a group with acute gastritis, consisting of 50 patients, and a group of chronic gastritis, comprising 49 patients. This division was based on the density of the mononuclear cell infiltrate depicted during microscopical analysis of gastric mucosa samples.

Mean age did not significantly differ between the two study subgroups and the controls, the highest mean age being identified among patients with a microscopic diagnosis of chronic gastritis: 11.38 ± 4.18 years for subjects diagnosed with acute gastritis, versus 13.02 ± 3.32 years for those diagnosed with chronic gastritis, and 12.32 ± 3.24 years in the control group, *p* = 0.13. Nevertheless, the female/male sex ratio was similar between the three groups. A significant, higher prevalence of chronic gastritis was identified among children coming from a rural background (*p* = 0.01). Considering that 32 out of the 39 patients (82.05%) diagnosed with *H. pylori* also had microscopic characteristics of chronic gastric inflammation, this result is expectable. The comparison of the demographic characteristics of the control group and the two study subgroups is exhibited in Table 3.

**Figure 4:**
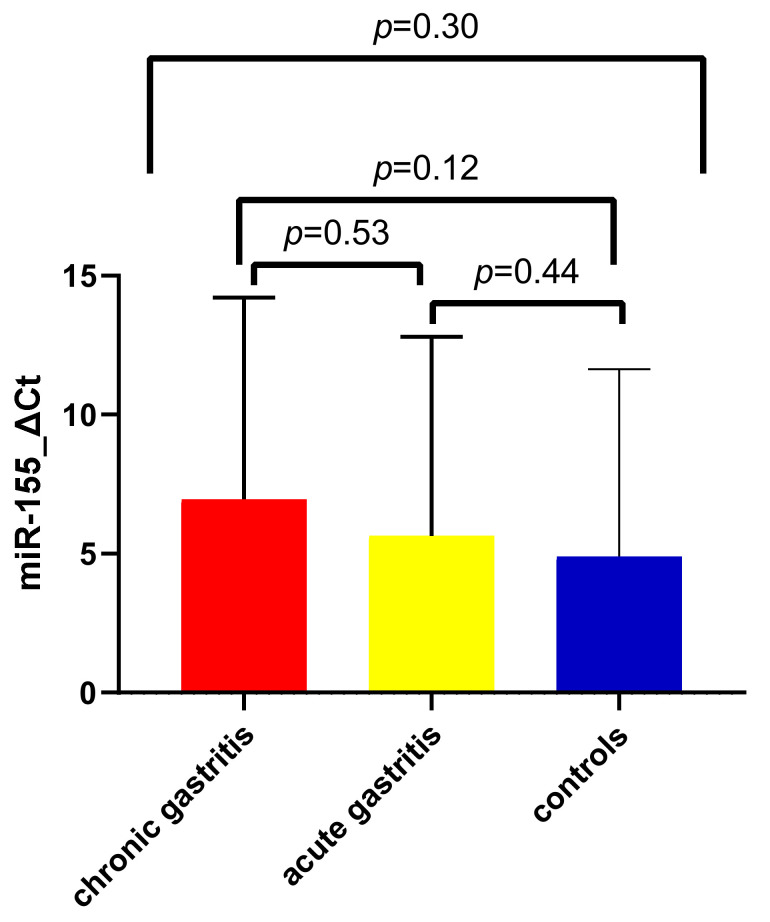
MiR-155 expression comparison between patients with acute gastritis, chronic gastritis, and control group.

Figure 4 illustrates the distribution of ΔCt values of miR-155 among controls, children with acute gastritis, and those with chronic gastritis. Mean values were increased in acute gastric inflammation (5.64 ± 7.15 SD) as opposed to the control group (4.90 ± 6.72 SD) and were the highest among those with chronic gastric inflammation (6.95 ± 7.27 SD). However, no significant difference was found after applying the Kruskal–Wallis test for the three groups (*p* = 0.3), nor after applying the Mann–Whitney test for individual comparison of mean expression between children with acute and chronic gastritis with controls, and among each other (*p* = 0.44, *p* = 0.12, and *p* = 0.53, respectively).

**Figure 5:**
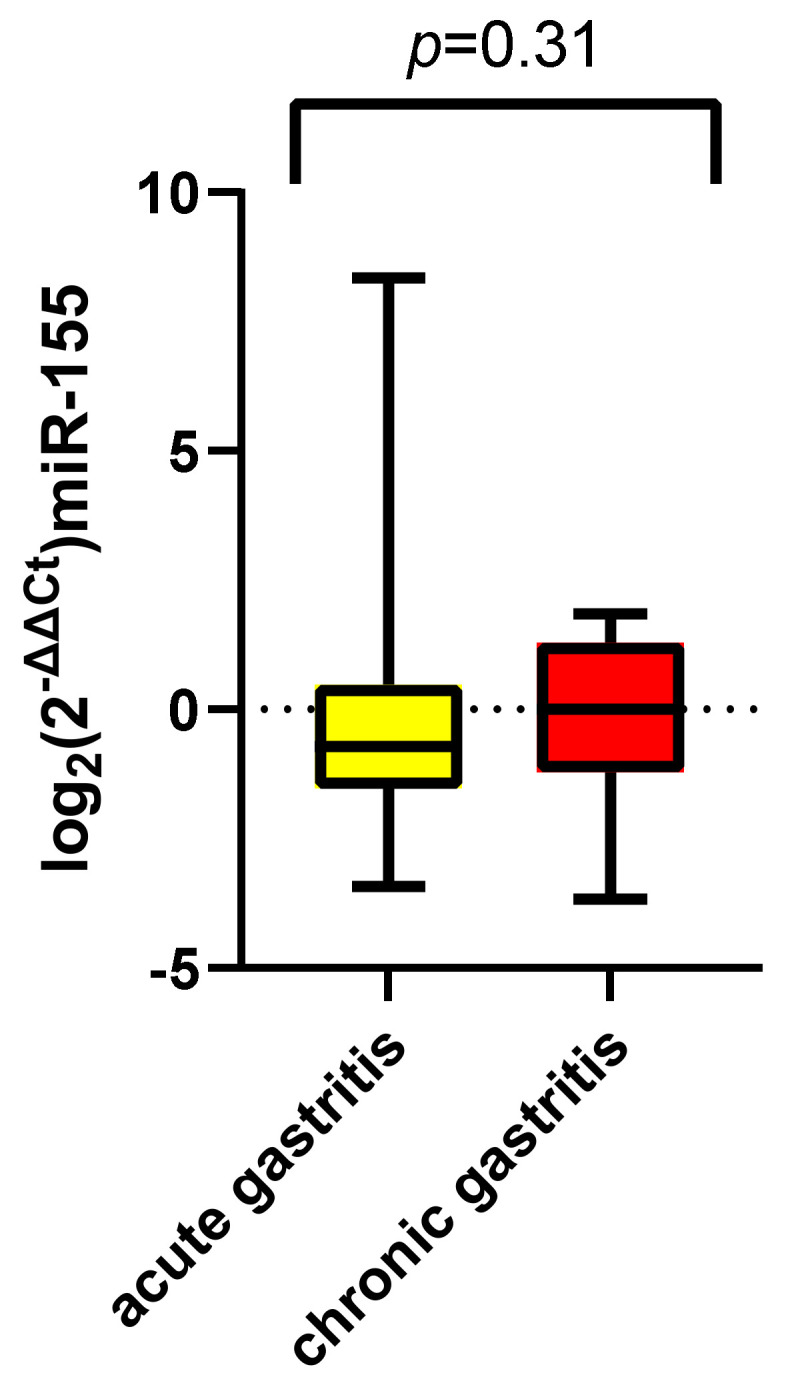
Log_2_(2^−ΔCt^) miR-155 comparison between patients with acute and chronic gastritis.

A comparison of the two study groups was later conducted after the calculation of log_2_(2^−ΔΔCt^) values. Figure 5 illustrates a higher distribution of the values among the acute gastritis group (−0.29 ± 2.45 SD), but with mean values similar to the chronic gastritis group (−0.20 ± 1.72 SD, *p* = 0.31).

### 3.4. Expression of miR-155 in Association with Gastritis Severity

Gastritis severity was assessed in accordance with the updated Sydney criteria, after taking into consideration the type of inflammatory infiltrate described, and its subsequent degree of activity. Most of the study group patients were suffering from mild gastritis (47 patients), while moderate gastritis was identified in 32 patients and severe gastritis in 20 subjects. There were no significant disparities between the control group and the three severity-dependent subgroups in terms of age, sex, and urban/rural background.

**Figure 6:**
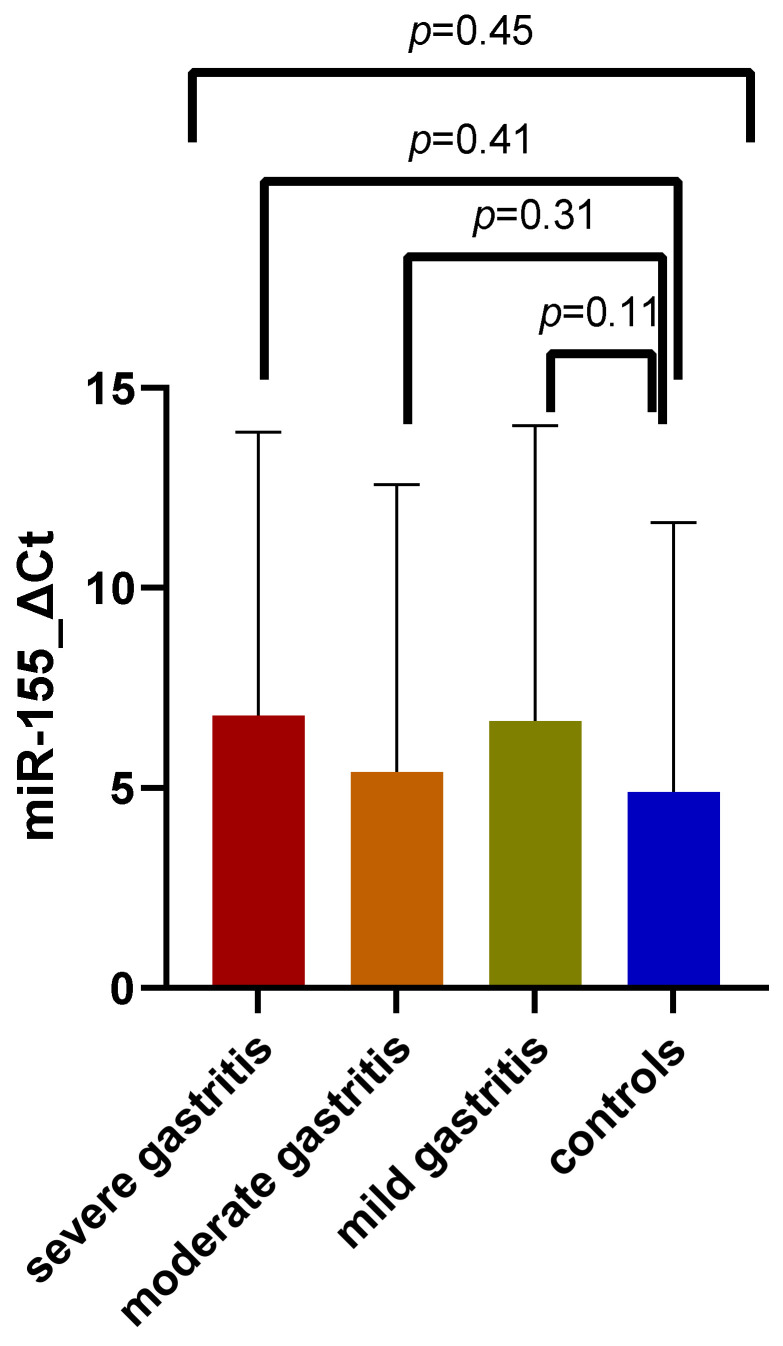
MiR-155 expression comparison between patients with severe gastritis, moderate gastritis, mild gastritis, and control group.

The distribution of miR-155 expression among the three study subgroups, as well as the overall comparison of these results, can be visualized in Figure 6 (*p* = 0.45). The highest expression was encountered among patients with severe gastritis (6.82 ± 7.07 SD), but the increase in mean ΔCt values was not dependent upon the severity, as lower mean values were encountered among the moderate gastritis group (5.40 ± 7.17 SD) than in patients with mild gastritis (6.67 ± 7.37 SD). There was no significant variation of mean values with gastritis severity, and no significant discrepancy between study subgroup patients and controls (mean value: 4.90 ± 6.72 SD).

**Figure 7:**
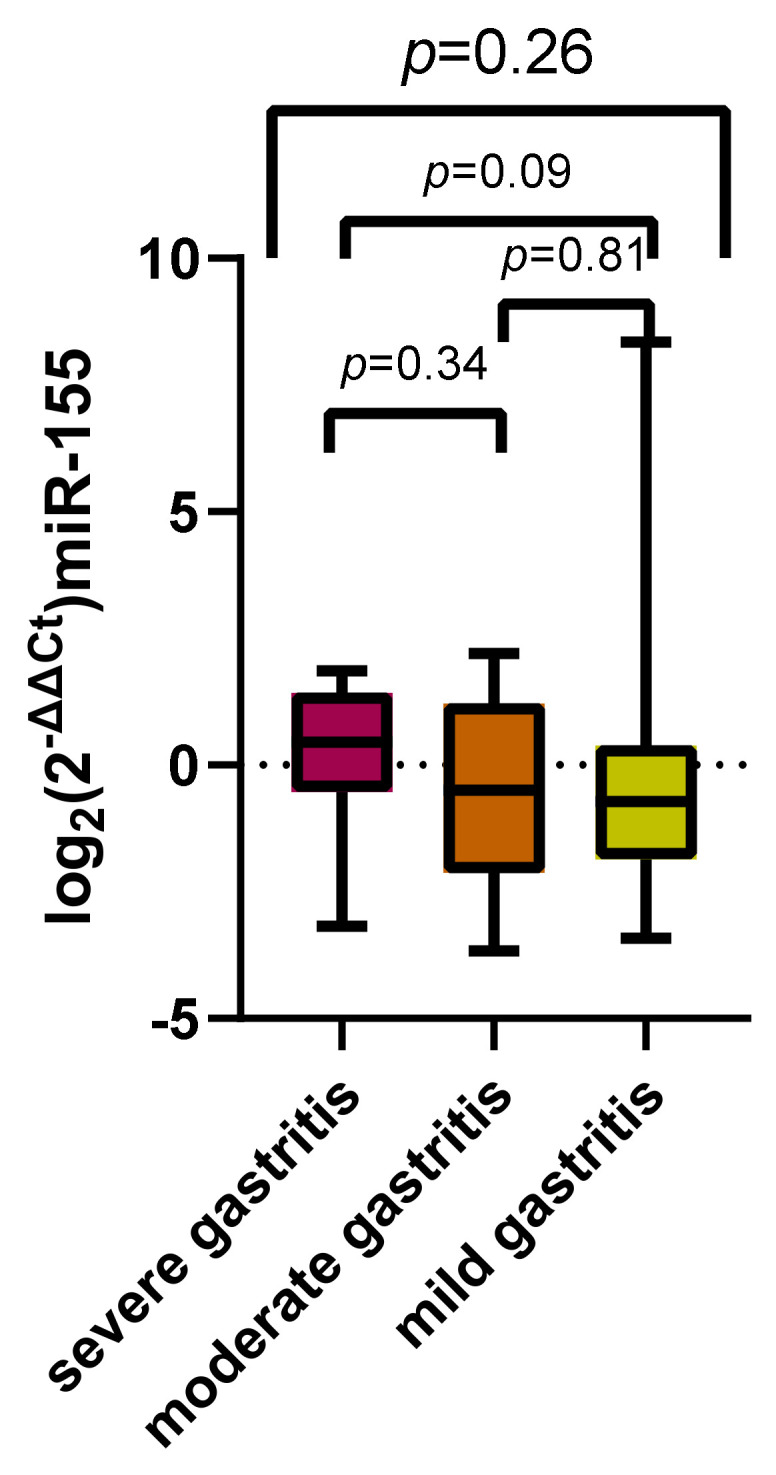
Log_2_(2^−ΔCt^) miR-155 comparison between patients with severe, moderate, and mild gastritis.

Similar results were obtained after applying the 2^−ΔΔCt^ method, and the values obtained did not present significant discrepancies after applying the Kruskal–Wallis test (Figure 7, *p* = 0.26).

## 4. Discussion

The quest for novel biomarkers in the diagnosis of gastritis, preneoplastic, and neoplastic gastric lesions has led to the discovery of several non-invasive serological tests, which can envision the potential unfavorable evolution of precancerous gastric conditions and/or detect early gastric cancer [28,29,30]. However, few studies have sought the individual or cumulative biomarker role of depictable serological/tissue biomarkers in gastric inflammation, which represents the initial, main histological anomaly.

MiRNAs are among the molecules with an expression change that is very sensitive to microscopic alteration of gastric mucosal integrity. Most studies have, so far, investigated their behavioral pattern in relation to *H. pylori* infection, with miRNAs acting as fine modulators of the immune response triggered by the pathogen [12,20,31,32]. Expression alteration seems not to be definitive, with one study proving that eradication of *H. pylori* infection seems to restore expression of particular miRNA molecules to normal [20]. MiR-155, one of the most intensely researched miRNAs, seems to be one of the miRNAs with a key role in the modulation of signaling pathways. *H. pylori* infection leads to upregulation of miR-155, through nuclear factor–κB (NF-κB) and activator protein-1 (AP-1) pathway activation [19]. MiR-155 is essential for proper immune response as a result of *H. pylori* infection, with one experimental study proving that miR-155 deficient mice infected with the aforementioned pathogen fail to produce an adequate Th1 and Th17 response, and consequently exhibit lower chances of developing preneoplastic conditions such as gastric atrophy and intestinal metaplasia [33]. Furthermore, miR-155 limits excessive inflammatory response by tampering with the release of interleukin-8 (IL-8) and growth-related oncogene-alpha [19].

Given the acknowledged role of miR-155 in the modulation of *H. pylori*-triggered immune response and inflammation, the present study tried to identify whether tissue expression of this particular miRNA is influenced by *H. pylori* infection-triggered gastric inflammation in children. Pediatric literature data regarding the role of miRNA molecules in various disorders are scarce and a possible association between miRNAs and *H. pylori* infection has so far been investigated in only three studies, with only one of them enrolling children with *H. pylori* gastritis [23,34,35]. This small-scale study, conducted by Cortes-Marquez et al., compared expression alteration of miR-155 and miR-146a in a pediatric, adult, and animal model population diagnosed with *H. pylori* and non-*H. pylori* gastritis. Upregulation of both miR-155 and miR-146a was found in human subjects diagnosed with *H. pylori* gastritis, as opposed to the study groups with gastric inflammation, in which the bacterial infection was absent. This expression variation was not significant in children, although it was more prevalent than in adults. A particularity of the study was the longitudinal evaluation of miR-155 and miR-146a expression in a *Meriones unguiculatus* population, at 3, 6, 12, and 18 months after inoculation of *H. pylori* infection. With chronological progression from infection inoculation, a prevalent, progressive upregulation of miR-146a expression was noted, together with a mild increase in miR-155 expression [23]. The study proved not only the additional role that *H. pylori* poses on miRNA expression alteration in patients with gastric inflammation, but also the importance of infection persistence for notable changes in miRNA molecules. Thus, the unsignificant change in miR-155 expression in association with *H. pylori* infection in our study might be explained by a lack of prolonged exposure to the pathogen, which is expectable at pediatric ages. Still, upregulation of miR-155 expression was also noted in our study, the expression difference from controls being greater in those children diagnosed with *H. pylori* gastritis.

The other two pediatric studies enrolling patients with non-gastric pathologies proved that *H. pylori* infection influences miRNA expression. Overexpression of miR-32-5p was discovered in association with *H. pylori*-induced pediatric enteritis, while downregulation of miR-204 was identified in children with pulpitis and *H. pylori* infection [34,35]. Both studies assessed non-invasive miRNAs, depicted from serum and/or saliva, but none of them provided any information about histopathological changes nor correlation with microscopic anomalies of the gastric mucosa [34,35]. Still, the studies raised the question whether *H. pylori* infection alone or the concomitant presence of an inflammatory condition of the gastrointestinal tract modifies miRNA expression.

One adult study concluded that *H. pylori* infection and chronic treatment with proton pump inhibitors can alter miRNA expression in subjects with gastritis, as opposed to NSAIDs or aspirin given as platelet aggregation inhibitors. Two miRNAs were analyzed, namely miR-155 and miR-223, which both presented significant expression augmentation in relation to *H. pylori* infection. However, eradication of the infection produced a significant decrease in the expression of miR-223 only [36]. In the context of *H. pylori* infection, the involvement of miRNAs such as miR-133a in methylation processes (which essentially contribute to the development of gastric cancer cell lines) has been documented, as well as the reversibility of this effect after infection eradication [37]. Thus, further research is necessary to evaluate the sole, prolonged impact of *H. pylori* infection on miRNA expression, as well as the potential reversibility of this phenomenon in association with infection eradication.

Other authors (Isomoto et al.) have tried to determine the expression variation of multiple miRNAs in relation to the secretion of four inflammatory cytokines, TNF-α, IL-1β, IL-6, and IL-8, in the context of *H. pylori* infection. They conducted an extensive study on 29 different miRNA molecules and identified a significant association between the messenger RNA (mRNA) levels of at least one of the cytokines and 17 different miRNA types [38]. In most (15) of these 17 miRNAs, a significant correlation between altered expression, chronic inflammation, and severity of atrophic gastritis (established in conformity with Sydney classification) was also reported [38]. Cortes-Marquez et al. also concluded that an increase in miR-155 expression was proportional to gastritis severity [23]. Similar to these studies, we noted progressive augmentation of miR-155 expression after applying the 2^-ΔΔCt^ method in children diagnosed with chronic gastritis within our research, but without significant discrepancy from patients with mild or moderate gastritis. Subjects diagnosed with severe gastritis accounted for only approximately 20% of our entire study group subjects, but these findings suggest that severe pediatric gastritis might, in time, lead to important variations of miR-155 expression.

The ability of miR-155 to predict the persistence of inflammation and potential evolution towards preneoplastic changes of the gastric mucosa has been highlighted in multiple studies [13]. Petrocca et al. reported augmentation of the expression of seven different miRNAs, including miR-155, in relation to chronic gastritis [39]. Alkaline water intake seems to also lead to an ascendant variation of miR-155 expression in relation to chronic gastric inflammation [40]. The involvement of miR-155 in Correa’s cascade of gastric cancer has been highlighted by a gradual increase in its expression, from the stage of non-atrophic chronic gastritis to atrophic gastritis and gastric cancer [41]. Furthermore, important miR-155 expression upregulation was identified in gastric mucosa-associated lymphoid tissue (MALT) lymphoma when compared to chronic gastric inflammation [42]. Similar overexpression of miR-155 was also reported in association with gastric diffuse large B-cell lymphoma [43]. Contrary to previous findings, our results did not show a consistent variation of miR-155 expression in relation to chronic gastritis, without significant discrepancy between normal gastric mucosa and acute gastric inflammation. Moreover, we identified preneoplastic lesions in only 2 patients from our entire study population, who we decided to exclude due to their small number, which would have raised into question the statistical validity of conducting comparisons with strikingly larger groups. Considering the paucity of preneoplastic lesions identified at pediatric ages, assessing the utility of miRNAs in relation to these conditions is extremely challenging.

The present study brings insights into the role of miR-155 expression in childhood and adolescence gastritis and adds novelty to the literature data by extending the research beyond *H. pylori* infection and including an important number of patients with gastritis of different etiologies. This is also the first pediatric study which assessed a possible correlation between miR-155 and gastritis severity, dependent upon the inflammatory infiltrate, as suggested by the Sydney classification. Moreover, our study also included a control group, which helped with a better understanding of miR-155 expression change from normal gastric mucosa towards local inflammatory modifications. Although upregulation of miR-155 in relation to *H. pylori* gastritis or chronic gastric inflammation was insignificant, the study proves that changes in miRNA expression occur in childhood, from the incipient stages of gastric inflammation. The lack of statistically significant results might be explained by the relatively short time of *H. pylori* infection exposure and the small number of patients with chronic gastritis encountered within our study. Therefore, continuous follow-up of miR-155 expression might reveal potential evolution towards preneoplastic adverse events.

The lack of miR-155 expression follow-up after *H. pylori* eradication is one of the limitations of the current research. Furthermore, our study did not seek to analyze a possible correlation with serum miR-155 expression and did not compare the findings with an adult population. Non-invasive miRNA depiction represents an actual trend, as they represent potentially comfortable diagnostic tools, but some authors contest their validity over tissue miRNAs, due to the fact that their expression level is greatly influenced by other circulating blood cells, diet, exercise, or even asymptomatic infections [44]. Thus, the utility of serum miRNAs is still put into question and an association with their tissue homologues requires further research.

## 5. Conclusions

MiR-155 expression is not greatly influenced by pediatric gastritis, according to our study. *H. pylori* infection, chronic gastric inflammation, or severe gastritis produced an upregulation of miR-155, but these results were not significant. Given the short time of *H. pylori* infection exposure, as well as that of the gastric inflammation evolution, specific to pediatric ages, dynamic evaluation of miR-155 might enlighten its long-term prognostic value. Still, further evaluation of multiple miRNAs in relation to childhood and adolescent gastritis and expanding the research on wider populations might represent important steps towards establishing the diagnostic and prognostic utility of these biomarkers, given the scarce pediatric literature data on the subject.

## Figures and Tables

**Table 1:** Demographic characteristic comparison of controls versus subjects with inflammatory modifications of the gastric mucosa.

Parameter		Control Group (*n* = 93)	Gastric Inflammation (*n* = 99)	*p* Value
Age-years (mean ± SD) ^a^		12.32 ± 3.24	12.19 ± 3.85	*p* = 0.09
Sex (%) ^b^	Female	26.56	30.20	*p* = 0.6, OR = 1.16 (CI: 0.67–2.03)
	Male	21.87	21.35	
Background ^b^	Urban	29.16	17.70	*p* < 0.01, OR = 2.89 (CI: 1.57–5.29)
	Rural	19.27	33.85	

Legend: CI—confidence interval, OR—odds ratio, SD—standard deviation; ^a^—Mann–Whitney test was used; ^b^—chi-square test was used.

**Table 2:** Demographic characteristic comparison of controls versus subjects with *H. pylori* gastritis and non-*H. pylori* gastritis.

Parameter		Control Group (*n* = 93)	*H. pylori* Gastritis (*n* = 39)	Non-*H. pylori* Gastritis (*n* = 60)	*p* Value
Age-years (mean ± SD) ^a^		12.32 ± 3.24	12.85 ± 3.39	11.77 ± 4.10	*p* = 0.48
Sex (%) ^b^	Female	26.56	11.45	18.75	*p* = 0.81
	Male	21.87	8.85	12.50	
Background ^b^	Urban	29.16	3.64	14.06	*p* < 0.01
	Rural	19.27	16.66	17.18	

Legend: SD—standard deviation; ^a^—Mann–Whitney test was used; ^b^—chi-square test was used.

**Table 3:** Demographic characteristic comparison of controls versus subjects with acute gastritis and chronic gastritis.

Parameter		Control Group (*n* = 93)	Acute Gastritis (*n* = 50)	Chronic Gastritis (*n* = 43)	*p* Value
Age-years (mean ± SD) ^a^		12.32 ± 3.24	11.38 ± 4.18	13.02 ± 3.32	*p* = 0.13
Sex (%) ^b^	Female	26.56	15.62	14.58	*p* = 0.83
	Male	21.87	10.41	10.93	
Background ^b^	Urban	29.16	12.5	5.20	*p* = 0.01
	Rural	19.27	13.54	20.31	

Legend: CI—confidence interval, OR—odds ratio, SD—standard deviation; ^a^—Mann–Whitney test was used; ^b^—chi-square test was used.

## Data Availability

Not applicable.
